# Determinants of mortality among neonates admitted to neonatal intensive care unit at public hospitals, in the Somali region, eastern Ethiopia: unmatched case-control study

**DOI:** 10.11604/pamj.2024.48.97.34341

**Published:** 2024-07-11

**Authors:** Zemenu Shiferaw Yadita, Elias Balcha, Semehal Haile Yohannes, Liyew Mekonen Ayehubizu

**Affiliations:** 1 Department of Reproductive Health and Population Studies, School of Public Health, College of Medicine and Health Sciences, Bahir Dar University, Bahir Dar, Ethiopia,; 2 Department of Nursing, College of Medicine and Health Sciences, Dembi Dollo University, Dembi Dollo, Ethiopia,; 3 Department of midwifery, College of Medicine and Health Sciences, Jigjiga University, Jigjiga, Ethiopia,; 4 Department of Public Health, College of Medicine and Health Sciences, Jigjiga University, Jigjiga, Ethiopia

**Keywords:** Neonatal mortality, determinants, neonatal intensive care unit, Somali region, Ethiopia

## Abstract

**Introduction:**

neonatal death is a global issue in both developed and developing countries. Evidence on determinants of neonatal mortality is scarce, particularly in the pastoralist and agro-pastoralist areas of Ethiopia, including the Somali region. This study aimed to identify the determinants of mortality among neonates admitted to neonatal intensive care units at selected public hospitals, in the Somali region, eastern Ethiopia.

**Methods:**

an unmatched case-control study design was employed from January 1^st^-30^th^, 2020. The total sample size was 312 neonates (156 cases and 156 controls) who were admitted to the neonatal intensive care unit from January 2018 to December 30^th^, 2019. A simple random sampling technique was used to select neonates. The data were collected by a structured checklist. Data entry and analysis were done by EpiData version 4.6 and SPSS version 23, respectively. Binary logistic regression using bivariate and multivariable analysis was done to identify determinants. Odds ratios were computed at 95% CI and a P value <0.05 was considered statistically significant.

**Results:**

a total of 310 neonates with complete medical charts (155 cases and 155 control) were included in the study. Lack of antenatal care (AOR 4.71; 95%CI: 1.41-15.75), rural residence (AOR=8.38; 95%CI: 2.22-31.69), premature rupture of membrane (AOR=4.29; 95%CI: 1.21-15.19), five-minutes APGAR score below-seven (AOR=9.87; 95%CI: 2.30-42.33), prenatal asphyxia (AOR=14.71; 95%CI: 2.79-77.33) and length of hospital stay ≤3 days (AOR=15.09; 95%CI: 2.89-78.62) were significantly associated with neonatal mortality.

**Conclusion:**

this study identifies rural residence, lack of antenatal care, premature rupture of membranes, low APGAR scores, prenatal asphyxia, and short hospital stays as determinants. Hence, improving access to basic health services such as antenatal care and early screening for pregnant mothers and newborns is critical. By prioritizing these interventions, healthcare systems can effectively work towards reducing neonatal mortality rates, ultimately improving the health outcomes of infants and their mothers.

## Introduction

Neonate is a child from birth to twenty-eight days of life, and the death of this period is neonatal death. It can be early neonatal death if it happens during the beginning of seven days. Late neonatal death if the death of a neonate occurs between seven to twenty-eight days of life [1]. Globally, 2.5 million neonates died; accounting for 47% of all under-five mortality worldwide [2]. Around 70% of neonatal deaths arise in only two regions, Africa and Southeast Asia. Sub-Saharan Africa region accounts for more than half of the deaths every year registered in Africa [3-5]. Ethiopia is one of the countries with higher neonatal deaths, and most of the deaths occur in the early neonatal. Neonatal death accounts for 42% of the under-5 mortalities in the country. A study indicated that neonatal death was 28 per 1000 live births period. Since 2000 neonatal mortality has fallen by 39% with an annual reduction rate of 2.2% [6-8]. The neonatal death or unfavorable outcome is due to negative maternal factors, fetal factors, and maternal obstetrics. These factors are preventable by preventing maternal infection, improving maternal nutrition and neonatal complications; and it can save around one-third of the death [2,9].

Several studies conducted in Nepal, Bangladesh, and Africa have found that maternal age less than 20 and older than 35 years were significantly associated with Neonatal death [10-13]. In Ethiopia, maternal age < 20 years was significantly associated with neonatal mortality [14]. Maternal residence in rural areas was another determinant of neonatal mortality [15,16]. The sex of neonates was one of the predictors for neonatal death, i.e. male neonates were high likely to die than female neonates [5,12,17,18]. On the other hand, neonates with less than average weight were more likely to die than those having average weight or more [5,11,14,16,19-21]. Appearance, Pulse, Grimace, Activity and respiration (APGAR) score at 1^st^ minute and 5^th^ minute was another variable associated with neonatal mortality conducted in different studies [5,21-23].

Mothers who become pregnant more than five times were found that significantly associated with neonatal mortality [5,8,10,11,14,16,21]. Lack of antenatal care (ANC) was a significant determinant of neonatal death as indicated in different studies conducted [8,14,17,24,25]. Studies conducted in neonatal intensive care unit (NICU) mothers who were singleton pregnant had a higher risk of loss of neonates than multiple [8,10,26]. A study shows that mothers who had complications of pre-eclampsia [11] and premature ruptures of the membrane were more at risk of losing their neonates than their counterparts [27].

Mode of delivery was one of the variables which indicated the risk of neonatal death. Studies show that giving birth through the cesarean section was associated with neonatal death [8,24]. A study in Goma Brazil found that gestational age < 37 has increased neonatal mortality [10,13,15,17,18,21]. Studies from Cameroon and Uganda showed that a higher rate of death was observed among neonates who didn't begin breastfeeding within an hour [8,28]. The risk of death in the first week of life is significantly associated with neonatal mortality [25,29,30].

Medical complications were affecting neonatal life: a facility-based study indicated that neonates who had respiratory distress syndrome (RDS) [15,21,29], prenatal asphyxia [8,18,21,24] neonatal sepsis or infection were associated with neonatal mortality [15,28,29]. Other studies showed neonatal hypothermia and hyperthermia [24], and meconium aspiration were determinants of neonatal mortality [8] and meconium aspiration were determinants of neonatal mortality [29]. A study done in Gitew district hospital in Ghana found that neonates born from mothers who had hypertension, HIV, diabetes mellitus, and anemia were, more likely to die than others [5].

Although there are several studies on determinants of neonatal death, there is very scarce evidence related to neonatal death in the pastoralist regions of Ethiopia, particularly the Somali region. Hence, it was very crucial and essential to identify determinants of neonatal mortality. The objective of this study was to determine the determinants of neonatal death, in the Somali regional state, of eastern Ethiopia.

## Methods

**Study design, setting and period:** an unmatched hospital-based case-control study design was conducted from January 1^st^-30^th^, 2020 to identify determinants of neonatal mortality among neonates who were admitted to the NICU of a selected public hospital, in Jigjiga, Somali region, eastern Ethiopia. The Somali Regional State, situated in the eastern and southeastern reaches of Ethiopia, is characterized by its pastoralist and agro-pastoralist communities. Despite its rich cultural heritage, the region grapples with significant challenges, notably the limited accessibility to essential healthcare services, particularly for mothers and children.

**Population, sample size determination and sampling procedure:** all births in the neonatal period at public hospitals, in the Somali region were the source population. The study population was all neonates who were admitted, died and survived from January 1^st^, 2018 to December 30^th^, 2019, at the public hospital of Jigjiga town, Somali region.

**Inclusion criteria:** for cases, all neonates who were admitted, and died in the hospitals were included and for controls, all neonates who were admitted and survived were included.

**Exclusion criteria:** all neonates with incomplete records were excluded.

The sample size was determined using double population proportion; considering the proportion of preterm among cases (61.4%), the proportion of preterm among controls (12.4%), AOR (3.37), ratio (1:1), a 95% confidence interval and power of the study (80%). Several factors were considered in the sample size determination. However, the maximum sample size was found by taking preterm birth as a factor. The final sample size was 312 (156 cases and 156 controls) [6]. Of a total of five eligible public referral hospitals, Sheik Hassen Yebere Referral Hospital was selected by lottery method. A total of 1439 neonates were admitted to the NICU of Sheik Hassen Yebere referral hospital. Then 156 cases and 156 controls were selected by computer-generated simple random sampling.

**Study variables:** the dependent variable was neonatal death. The independent variable includes; socio-demographic characteristics (age at admission, gestational age, sex of neonate, weight of neonate, maternal age, residency), obstetric and gynecological variables (ANC follow-up, gravidity, parity, mode of delivery, multiple pregnancies, PROM, preeclampsia, abruption placenta, breastfeeding initiation, APGAR score), maternal medical complication (HIV, anemia, hepatitis B, hypertension) and neonatal medical complication (RD, sepsis, jaundice, hypothermia, PNA).

**Data collection:** the medical records (chart) were first checked and the data was extracted by using a checklist. The checklist was developed from works of literature and it contains; socio-demographic characteristics, obstetric and medical-related factors, and the neonate-related conditions. The checklist was pretested on 5% of the sample size at Kebri Dahir Referral Hospital. Data collectors were intensively trained to ensure data quality.

### Measurement

***Low APGAR score:*** a neonate with an APGAR score of <7 [30].

***Neonatal death:*** death of neonate until 28 days of delivery [1].

***Cases:*** newborns aged from 0 to 28 days admitted and died.

***Controls:*** newborns aged from 0 to 28 days admitted and lived.

**Statistical analysis:** the data underwent thorough cleaning procedures to address inconsistencies and missing values, with necessary modifications made as required, before being inputted into Epi-data version 4.6 and subsequently exported to SPSS Version 23 for analysis. Descriptive statistics, including frequencies, percentages, means, and standard deviations, were utilized, while binary logistic regression was employed to identify the determinants of neonatal mortality. Initially, bivariate logistic regression analysis was conducted to identify variables with a p-value of less than or equal to 0.2, which were then considered as candidates for multivariable regression analysis. Subsequently, multivariable logistic regression analysis was performed to identify determinants, considering a significance threshold of P-value below 0.05, with adjusted odds ratios (AOR) calculated along with 95% confidence intervals (CI) to measure association. The goodness of fit of the model was assessed through Hosmer-Lemeshow tests, while collinearity was examined via variance inflation factor (VIF) and tolerance tests.

**Ethical consideration:** letter of ethical approval was obtained from Jigjiga University Research Ethics Review Committee. Further permission was obtained from the medical director's office and pediatrics department of Sheik Hassen Yebere Referral Hospital for utilization of medical records. Confidentiality was maintained by excluding names or other personal identification from the data collection record sheet.

## Results

**Socio-demographic characteristics of the study participant:** a total of 156 (cases) and 156 (controls) neonates were included in the study. Among 156 neonates who were taken as cases, 78 (50.0%) had mothers aged between 21-34 years old. The mean age of mothers was 28.5±13.7 years (IQR). In more than half of the cases 89 (57.1%) mothers of the neonates were rural residents. About 147 (94.2%) of cases and 136 (87.2%) of controls were in the early neonatal period. The Mean age of neonates was 2.5±4.6 days (SD). Concerning the sex of neonates 79 (50.6%) and 77 (49.7%) cases were male and female respectively. Besides that, more than half 85 (58.4%) of the controls weighed between 2500-4500gm and 65 (41.7%) of case weight 1501-2499. The mean weight was 2300 ±1200gram (IQR) (Table 1).

**Table 1:** socio-demographic characteristics of mothers and neonates admitted to the NICU of a public hospital in Somali region, eastern Ethiopia 2020 (N=312)

Variable	Category	Cases # (%)	Controls # (%)	Total # (%)
Age of Mothers	≤20	46(29.5)	40(25.6)	86(27.6)
	21-34	65(41.7)	78(50.0)	143(45.8)
	≥35	45(28.8)	38(24.4)	83(26.6)
Place of Residence	Rural	89(57.1)	64(41.0)	153(49.0)
	Urban	67(42.9)	91(59.0)	159(51.0)
Age of Neonate	0-6day	147(94.2)	136(87.2)	281(90.7)
	7-28day	9(5.8)	20(12.8)	29(9.3)
Weight	≤1500	42(27.5)	8(5.1)	50(16.0)
	1501-2449	65(41.7)	57(36.5)	122(39.1)
	2500-4500	43(28.9)	85(58.4)	140(44.9)
Sex of neonates	Male	93(59.6)	77(49.7)	170(54.5)
	Female	63(40.4)	79(50.6)	142(45.5)

NB: # is frequency

**Obstetrics characteristics of mothers of the neonates:** ninety-nine (63.5%) of mothers of controls had ANC follow-up at least one visit and 84 (53.8%) of mothers of cases had no ANC follow-up. Regarding, the number of pregnancies 133 (85%) of mothers of cases had become pregnant less than or equal to five times. Moreover, nearly half (75 (48.7%)) of control neonates were delivered from multiparous mothers. The majority of the cases were delivered from mothers with singleton pregnancies. Eighty-three (53.2%) mothers who had premature rupture of membrane lost their neonates. Of the total 156 cases, one hundred and fifteen (73.7%) gave birth through SVD (Table 2).

**Table 2:** obstetric/gynecological characteristics of mothers of neonates who were admitted to the NICU of a public hospital in Somali region, eastern Ethiopia 2020 (N=312)

Variable	Category	Case(n=156) %	Controls(n=156) %	Total # (%)
Gravidity	≤5	133(85.3)	135(86.5)	268(85.9)
	≥6	23(14.7)	21(13.5)	44(14.1)
Parity	Prim	67(42.9)	53(34.0)	120(38.5)
	2-4	56(35.9)	75(48.7)	131(42.0)
	≥5	33(21.2)	28(17.9)	61(19.5)
Multiple pregnancies	Yes	27(17.3)	12(7.7)	39(12.5)
	No	129(82.7)	144(92.3)	273(87.5)
PROM	Yes	83(53.2)	47(30.1)	130(41.7)
	No	73(46.8)	109(69.9)	182(58.3)
Preeclampsia	Yes	21(13.5)	14(9.0)	35(11.8)
	No	135(86.5)	142(91.0)	277(88.8)
Abruption Placenta	Yes	2(1.3)	3(1.9)	5(1.6)
	No	154(98.7)	153(98.1)	307(98.4)
Others	Yes	76(48.7)	88(56.4)	164(52.6)
	No	80(51.3)	68(43.6)	148(47.4)
Mode of delivery	SVD	115(73.7)	96(61.5)	211(67.6)
	C/S	36(23.1)	53(34.0)	89(28.5)
	Instrumental	5(3.2)	7(4.5)	12(3.9)
ANC follow up	Yes	72(46.2)	99(63.5)	171(54.8)
	No	84(53.8)	57(36.5)	141(45.2)

**Neonatal characteristics:** it is important to note that 67 (42.9%) of cases were delivered at a gestational age of ≤ 32 weeks. The mean gestational age of neonates was 36±6 weeks (inter-quartile range (IQR)). One hundred and thirty-five (86.5%) of cases scored an APGAR below 7 in the 1^st^ minute. Additionally, 97 (62.2%) of cases scored an APGAR below 7 at the 5^th^ minute. The majority, 125 (80.8%) of cases were not breastfed. It is important to note that, 145 (92.9%) of cases didn't feed within one hour. Seventy-five (48.1%) of cases stayed at the NICU for less than 3 days. However, controls who stayed at the NICU for more than 8 days were 73 (46.8%) (Table 3).

**Table 3:** neonatal characteristics of neonates admitted to the NICU of a public hospital in Somali region, eastern Ethiopia 2020 (n=312)

Variable	Category	Cases(n=156) %	Controls(n=156) %	Total n (%)
GA	≤32	67(42.6)	25(16.0)	92(29.5)
	33-37	58(37.2)	77(49.4)	135(43.3)
	38-42	31(19.9)	54(34.6)	85(27.2)
APGAR score at 1st min	<7	135(86.5)	87(55.8)	222(71.2)
	≥7	21(13.5)	69(44.2)	90(29.8)
APGAR score at 5th min	<7	97(62.2)	27(17.3)	124(39.7)
	≥7	59(37.8)	129(82.7)	188(60.3)
Breastfeeding	Yes	30(19.2)	153(98.1)	183(58.7)
	No	125(80.8)	3(1.9)	129(41.3)
Within one hour	Yes	11(7.1)	63(40.4)	74(23.7)
	NO	145(92.9)	93(59.6)	238(76.3)
After one hour	Yes	20(12.8)	89(57.1)	109(34.9)
	No	136(87.2)	67(42.9)	203(65.1)
Length of hospital stay in days	≤3	75(48.1)	22(14.1)	96(31.1)
	4-7	47(29.1)	61(39.1)	108(34.6)
	≥8	34(21.8)	73(46.8)	107(34.3)

**Figure 1:**
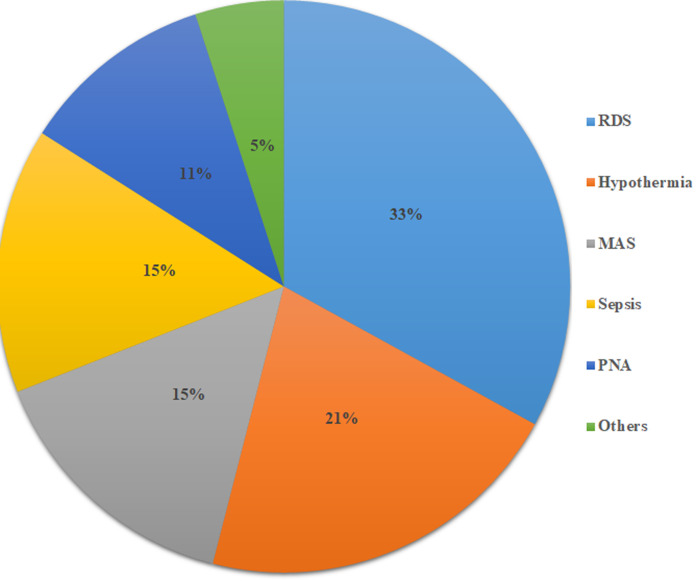
neonatal complications among neonates who were admitted to NICU of a public hospital in Somali region, eastern Ethiopia, 2020 (N=312); (Note: RDS-respiratory stress syndrome, PNA-perinatal asphyxia, MAS-meconium aspiration syndrome)

**Mothers and neonatal medical complications:** of all neonates who were included in this study, 30 (9.6%) of them were born from mothers who had experienced medical complications including hypertension, hepatitis B, and anemia. Regarding neonatal complications, more than one-third (33 103) of the neonates have developed respiratory distress syndrome (RDS) (Figure 1).

**Determinants of neonatal mortality:** initially, bivariate logistic regression analysis was done and crude odd ratios were computed. Twenty-two variables with a p-value of below 0.2 were identified as candidates for multivariable analysis. In the multivariable logistic regression analysis, rural residence, lack of at least one antenatal care visit, premature rupture of membrane, 5^th^ minute APGAR, prenatal asphyxia, and less than three days' hospital stay were significantly associated with neonatal mortality, considering p-value below 0.05 as cut off point.

The residence of the mother was associated with neonatal death. The odds of neonatal death among mothers who reside in rural areas were eight times more likely (AOR= 8.38, 95% (CI: 2.22-31.69) than their counterparts. Mothers who lack ANC follow-up were nearly five times more likely (AOR=4.71, 95% (CI: 1.41-15.75) to lose their neonates than mothers who had ANC follow-up. The odds of neonatal death among mothers who experienced premature ruptures of membrane (PROM) were four times more likely (AOR=4.29, 95 % (CI: 1.21-15.19) than mothers without experience of PROM. Neonates who were admitted with complications of perinatal asphyxia were fifteen times more likely (AOR=14.71, 95% (CI: 2.79-77.33) to die than neonates without perinatal asphyxia.

Neonates who had a 5^th^ minute APGAR score less than or equal to three were ten times (AOR=9.87, 95% (CI:2.30-42.33) more likely to die than neonates whose APGAR score greater or equal to seven. Regarding the hospital stay, neonates who stayed for less than three days were fifteen times more likely (AOR=15.09, 95% (CI:2.89-78) to die than neonates who stayed for more than three days, in the hospital (Table 4).

**Table 4:** bivariate and multi-variable logistic regression analysis results for factors associated with neonatal mortality in the NICU of a public hospital in Somali region, eastern Ethiopia, 2020 (n=312)

Variables	Category	Case	Control	COR with 95% CI*	AOR with 95% CI	P-value
Residence	Urban	67	92	1	1	
	Rural	89	64	1.87(1.19, 2.93) *	8.38(2.22-31.69) **	0.002
ANC follow up	Yes	72	99	1	1	
	No	84	57	2.09(1.33,3.29) *	4.71(1.41-15.75) *	0.012
PROM	Yes	83	47	2.66(1.67,4.25) *	4.29(1.21-15.19) *	0.024
	No	73	109	1	1	
5thmin APGAR	<7	97	27	7.93(4.68,13.44) *	9.87(2.30-42.33) **	0.002
	≥7	59	129	1	1	
Prenatal asphyxia	Yes	72	17	7.34(4.01-13.46) *	14.71(2.79-77.33) **	0.001
	No	84	139	1	1	
Length of hospital stay	≤3 days	75	22	7.67(4.07-14.43) *	15.09(2.89-78.62) **	0.001
	4-7 days	47	61	1.62(0.93-2.83) *	1.76(0.45-6.84)	
	≥8 days	34	73	1	1	

Significant at *p-value ≤0.05, **p-value≤0.01

## Discussion

This study aimed to determine neonatal mortality among neonates admitted to NICU at a Public Hospital in the Somali region, eastern Ethiopia. Analysis showed that neonatal mortality was significantly associated with place of residence. Neonates whose mothers reside in the rural were seven times more at risk of death than urban residents. This finding is consistent with studies conducted in the eastern Wollega, Jimma zone and systematic reviews done in Ethiopia [15,16,31]. This may be because women living in rural areas are less likely to get a nearby health facility and may not arrive on time for labor and delivery. This can put newborns at risk for death.

Lack of at least one ANC follow-up during pregnancy has increased the risk of neonatal mortality. This finding is supported by similar studies conducted in western and eastern Ethiopia [14,17]. It is also consistent with the study conducted in Afar and the Amhara region [24,32]. This is because mothers who had ANC have better access to early detection of medical and obstetrics complications. As a result, promoting ANC follow-up, establishing good maternal health care during ANC, and early postnatal period are very important for better maternal and neonatal health outcomes.

Neonates who scored less than seven APGAR at the fifth minute were eight times more likely to die compared to those neonates with APGAR above seven. This study is supported by pieces of evidence from eastern Ethiopia and Kenya [5,20,27]. This may be due to the lack of equipment for resuscitation and health professional's reluctance to register or assess the newborn according to the standards.

Premature rupture of the membrane was observed to increase the risk of neonatal death. This finding is consistent with the study conducted on the tertiary hospitals in Kenya [27]. Besides their consistency, this study is two times higher than the study conducted in northeast Ethiopia at a neonatal intensive care unit [9]. Premature rupture of the membrane can result in complications in the newborn and the mothers including sepsis, asphyxia, and meconium aspiration syndrome which may lead the newborn to prolonged stay in the hospital. Therefore, early detection and treatment of mothers who had premature rupture of the membrane were very important.

Neonates who had complications of perinatal asphyxia (PNA) had an increased risk of death than their counterparts. These results indicated that higher than the study conducted in southern Ethiopia, the Afar region and the Jimma Zone [8,24,31]. Similarly, neonates diagnosed with reparatory distress were more likely to die than others. This may indicate that neonates who had PNA during admission have a higher risk of death or poor prognosis than neonates who did not. Hence, strict follow-up of the mother during labor with partograph is imperative to reduce the risk of asphyxia.

The odds of neonatal death with meconium aspiration syndrome were four times more likely to die than those who never had [29]. Furthermore, neonates who stay in the hospital for less than or equal to three days had sixteen times the risk of death than neonates who stay from seven days until the end of the neonatal period. This result is consistent with studies conducted in the eastern part of Ethiopia [20,33]. A study from Jimma town also showed that neonates who stayed in the hospital for less than seven days had four times the risk of death [31]. This may be due to incomplete treatment and lack of basic care at the household level.

**Strength of study:** the strength of this study is that it employed a case-control study design that enabled the identification of multiple factors for neonatal death.

**Limitation of the study:** this study was based on secondary data (patient chart), it may not display all factors of neonatal mortality and missing neonatal deaths, particularly those charts of neonates with no written discharge summary and patient status.

## Conclusion

This study demonstrated that maternal residence in rural, lack of ANC follow up, PROM, 5^th^ minute APGAR score less than seven, PNA and length of hospital stay (less than 3 days) significantly associated factors with neonatal mortality. This study suggests, regularly ANC follow up, scaling up newborn care and early detection and treatment of mothers who had premature rupture of membrane. Increasing the availability of health facility in the rural areas, crating mobile case team service, and increases knowledge of about ANC is crucial.

### What is known about this topic

- *Premature rupture of membrane is increasing risk of neonatal death;*
- *Neonatal death is higher among those neonates with lesser APGAR score*.

### What this study adds

- *Lack of access for antenatal care (ANC) is a predictor factor for death of neonates in the pastoralist area;*
- *Shorter duration (less than three days) of hospital stay increases neonatal death;*
- *Neonates who come from hard to reach areas were more likely to die*.
